# Health professionals and the early detection of head and neck cancers: a population-based study in a high incidence area

**DOI:** 10.1186/s12885-016-2531-7

**Published:** 2016-07-12

**Authors:** Karine Ligier, Olivier Dejardin, Ludivine Launay, Emmanuel Benoit, Emmanuel Babin, Simona Bara, Bénédicte Lapôtre-Ledoux, Guy Launoy, Anne-Valérie Guizard

**Affiliations:** General Cancer Registry of Lille and its area, GCS-C2RC, F-59037 Lille, France; University Hospital of Caen, U1086 INSERM UCBN “Cancers & preventions”, F-14000 Caen, France; ERSM-Nord, F-59665 Villeneuve d’Ascq, France; Department of Otorhinolaryngology and Cervicofacial Surgery, University Hospital of Caen, F-14000 Caen, France; General Cancer Registry of Manche, Centre Hospitalier Public du Cotentin, F-50100 Cherbourg-Octeville, France; General Cancer Registry of Somme, Hôpital Nord, F-80054 Amiens, France; Pôle de Recherche, Centre Hospitalo-Universitaire de Caen, F-14000 Caen, France; General Cancer Registry of Calvados, U1086 INSERM UCBN “Cancers et preventions”, Centre F Baclesse, F-14000 Caen, France

**Keywords:** Early detection, Head and neck cancers, Cancers registry, Socio-economic factors, Stage at diagnosis, Uptake of healthcare, Health insurance, Epidemiology

## Abstract

**Background:**

In the context of early detection of head and neck cancers (HNC), the aim of this study was to describe how people sought medical consultation during the year prior to diagnosis and the impact on the stage of the cancer.

**Methods:**

Patients over 20 years old with a diagnosis of HNC in 2010 were included from four French cancer registries. The medical data were matched with data regarding uptake of healthcare issued from French National Health Insurance General Regime.

**Results:**

In 86.0 % of cases, patients had consulted a general practitioner (GP) and 21.1 % a dentist. Consulting a GP at least once during the year preceding diagnosis was unrelated to Charlson index, age, sex, *département*, quintile of deprivation of place of residence. Patients from the ‘quite privileged’, ‘quite underprivileged’ and ‘underprivileged’ quintiles consulted a dentist more frequently than those from the ‘very underprivileged’ quintile (*p* = 0.007).

The stage was less advanced for patients who had consulted a GP (OR = 0.42 [0.18–0.99]) - with a dose–response effect.

**Conclusions:**

In view of the frequency of consultations, the existence of a significant association between consultations and a localised stage at diagnosis and the absence of a socio-economic association, early detection of HNC by GPs would seem to be the most appropriate way.

## Background

In Europe, head and neck cancers (HNC) are the forth most common group of cancers among men with an estimated annual incidence of 109 900 cases and 52 300 deaths [1]. Among women these cancers are less common. France, especially in the north-west [2, 3], has an incidence rate amongst the highest in Europe [1], although it is constantly decreasing [4]. In France in 2012, the world standardised incidence rate of cancers situated in the lips, mouth and pharynx was 16.1 cases per 100 000 person-years (p-y) for men and 5.6 cases per 100 000 p-y for women. For the larynx, rates were 5.4 and 0.9 cases per 100 000 p-y respectively for men and women.

The main risk factors are tobacco and alcohol. Other risk factors have been identified or are suspected, including *Human Papilloma Virus* infection, a diet lacking in fruit and vegetables, exposure to carcinogens in some work environments, teeth in poor condition or *Human Immunodeficiency Virus* infection [5]. In addition, these cancers are strongly linked to socio-economic factors: there are more deaths from HNC in people with a lower level of education compared with people with a higher level [6]. The risk of developing one of the HNC is greater in those with low incomes, with a low level of education, or belonging to a poorer socio-professional category [7]. These social differences persist even after adjustment for tobacco and alcohol consumption and for dietary factors [8].

Only in 30 % of cases, HNC are diagnosed at a localised stage [9, 10]. Late diagnosis is associated with a lower survival rate : 5-year survival in patients diagnosed at an advanced stage is reduced by a factor of 2 to 4 compared with that for patients diagnosed at a localised stage [9–11].

Also, time from symptom onset to treatment can be long. A recent review showed that the patient delay varied from 3.5 to 5.4 months and professional delay from 14 to 21 weeks [12]. Professional delay depends essentially on multi-disciplinary patient management (oral rehabilitation, refeeding, etc.), and healthcare delivery factors. Some studies showed that these delays lead to tumour growth [13, 14], advanced stage [15], or even an increased risk of death for the patient [16].

The prognosis is therefore extremely poor, with a net 5-year survival of only 32 % in France [17]. This figure is considerably lower than that in other European countries [18] and has hardly improved over the last 15 years.

On top of this, aggressive treatment regimes following late diagnosis can lead to serious sequels that affect quality of life, notably through changes in phonation, respiration, nutrition and physical appearance [19].

In view of this, screening programmes or early diagnosis of these cancers should be a pressing concern in public health, particularly for oral cavity lesions, which are easily accessible on clinical examination. The last review of the literature by the Cochrane group highlighted the lack of studies that would enable an assessment of the efficacy and cost of a screening programme for oral cavity cancers [20]. However, it also recommended ‘opportunistic visual screening by trained dentist and oral health practitioners’, especially for smokers and patients who drink alcohol.

The French governmental cancer plan 2009–2013 [21] advocated early detection of oral cavity cancers. Following this, in spite of the absence of any scientific proof, probably to demonstrate a pro-active attitude, the National Cancer Institute (INCa) set up multimedia training for dentist (2009) and general practitioners (2010) to teach them how to detect suspicious lesions through an in-depth examination of the oral cavity in high-risk patients [22].

Few works have studied the health habits prior to HNC diagnosis among these patients. A study among HNC patients in a Medicare population showed that about 90 % had had at least one visit to a physician in the year prior to diagnosis [23]. Another study showed that 82 % of HNC patients had first visited a general practitioner and 12 % a dentist (Tromp [24]). In France, health habits prior to HNC diagnosis are unknown among these patients who are often in a socially fragile position linked to their addiction to tobacco and alcohol.

The aim of this study was to describe how people sought medical consultation during the year prior to HNC diagnosis and the impact of these consultations on the stage of the cancer at diagnosis.

## Methods

### Study population

Included in the study were patients over 20 years old, covered by the French National Health Insurance General Regime, with a diagnosis of epithelial infiltrating HNC reported between 1 January 2010 and 31 December 2010 (*N* = 342, Table 1). Head and neck cancer cases were comprised of the anatomic sites oral cavity, oropharynx, hypopharynx and larynx (*International Classification of Diseases for Oncology*, 3rd edition - ICD-O 3 codes : C01-C06, C09-C10,C12-C14 and C32).

**Table 1:** Patient and tumour characteristics and univariate analysis of tumour stage at diagnosis

	Stage I-II^a^		Stage III-IV^a^		p univariate	Total	
	N	%	N	%		N	%
Sex					0.209		
Male	75	27.5	198	72.5		278	81.3
Female	22	35.5	40	64.5		64	18.7
Age at diagnosis					0.220		
< 55 years	32	27.6	84	72.4		119	34.8
55–65 years	33	25.4	97	74.6		130	38.0
> =65 years	32	36.0	57	64.0		93	27.2
Charlson Index					0.290		
0	14	35.0	26	65.0		41	12.0
1 to 2	42	25.1	125	74.9		169	49.4
3 and over	41	32.0	87	68.0		132	38.6
*Département*					0.531		
Calvados	16	23.5	52	76.5		69	20.2
Manche	15	31.3	33	68.8		48	14.0
ZPL	43	32.6	89	67.4		135	39.5
Somme	23	26.4	64	73.6		90	26.3
Tumour site					<0.001		
Oral cavity	44	36.7	76	63.3		124	36.3
Oropharynx	13	15.3	72	84.7		85	24.8
Hypopharynx	4	6.5	58	93.5		63	18.4
Larynx	36	52.9	32	47.1		70	20.5
Deprivation quintile^b^					0.065		
Privileged 1	14	32.6	29	67.4		43	12.6
Quite privileged 2	11	23.9	35	76.1		47	13.8
Quite underprivileged 3	18	47.4	20	52.6		40	11.8
Underprivileged 4	22	30.6	50	69.4		74	21.8
Very underprivileged 5	32	23.9	102	76.1		136	40.0
GP consultation					0.110		
No	9	19.1	38	80.9		48	14.0
Yes	88	30.6	200	69.4		294	86.0
GP consultation (in 3 categories)					0.071		
No consultation	9	19.1	38	80.9		48	14.0
1 to 2 consultations	14	22.2	49	77.8		64	18.7
> =3 consultations	74	32.9	151	67.1		230	67.3
Dentist consultation					0.029		
No	70	26.2	197	73.8		270	79.9
Yes	27	39.7	41	60.3		72	21.1
Specialist consultation, non-ENT					0.131		
No	45	25.4	132	74.6		181	52.9
Yes	52	32.9	106	67.1		161	47.1
ENT specialist consultation					0.002		
No	71	25.4	208	74.6		286	83.6
Yes	26	46.4	30	53.6		56	16.4
Nurse consultation					0.646		
No	54	27.0	139	73.0		195	57.0
Yes	43	30.3	99	69.7		147	43.0

^a^for seven patients stage at diagnosis was unknown

^b^for two patients the quintile of deprivation was unknown

Abbreviations: *ZPL* area around Lille, *GP* general practitioner, *ENT* ear, nose and throat

The patients were taken from the cancer registries of the Calvados, Manche and Somme *départements* and the area around Lille (ZPL). These registries meet high-quality criteria : the completeness and data quality are regularly assessed by the *Comité National des Registres*. Patients with a prior invasive or *in situ* cancer (excepting basal-cell and squamous-cell skin tumours) were excluded from the study.

### Medical data

As part of a high resolution study, data were extracted from the medical files and included the patient’s date of birth, gender, address, comorbidities, date of diagnosis, the topography and morphology of the cancer according to the ICD-O 3, the clinical stage of the tumour at diagnosis (TNM stage from the International Union Against Cancer’s *TNM Classification of malignant tumors*, 7th edition) and the existence of a synchronous HNC (within a 6-month period).

Comorbidities were classified using the Charlson comorbidity index [25]. Patients were divided into 3 groups for comorbidity: 0 (no comorbidity), 1–2 (moderate comorbidity), 3 and over (severe comorbidity).

### Data regarding uptake of healthcare

For patients included in the study, data concerning healthcare uptake were supplied by the general regime of the national health insurance (ERASME database –Extraction, Research, Analysis and Medico-Economic monitoring) which covers 88 % of the French population [26]. Data extracted included dates of consultation and the speciality of the medical practitioner consulted. They also concerned the date of declaration of the referring doctor. Since 2004, each patient has to declare a referring doctor for the reimbursement of care in France. The referring doctor, usually a general practitioner, is the first medical practitioner contacted by the patient. He regulates access to specialist.

Patients who had declared a referring doctor in the 2 months preceding diagnosis or after diagnosis were considered to have no referring doctor before diagnosis.

Only consultations between 2 and 12 months before cancer diagnosis were taken into account. The codes of health professionals who had carried out consultations or procedures were categorised into the following groups: general practitioner (GP), dentist, Ear, Nose and Throat specialist (ENT specialist) and other specialist (non - ENT specialist) nurse.

### Socio-economic data

As there is no individual socio-economic data in the medical records, the socio-economic status of patients was evaluated by measuring that of their place of residence using a social deprivation index. The index used was the EDI [27]. This is based on both individual data from the European Union Statistics on Income and Living Conditions (EU-SILC) survey and aggregated data (at the IRIS level - *Ilots Regroupés pour l'Information Statistique*–, which is the smallest geographical unit for which figures are available) from the 2007 French national census carried out by INSEE, the national institute for statistics and economic studies. The IRIS for each patient was determined by the home address at the time the HNC diagnosis was made.

In our statistical analyses, we used the national quintile of this index.

### Data analysis

We tested for associations between qualitative variables using the chi-square test or Fisher’s exact test. Quantitative variables were described by median and 25th and 75th percentiles (Q1-Q3). In order to determine factors influencing the probability of seeking healthcare or factors leading to diagnosis at an advanced tumour stage (stage I – II vs III – IV), logistic regressions were used. Regarding the influence of a consultation with each type of health professional on tumour stage at diagnosis, multivariable models were used. Odds ratios (OR) were presented with their 95 % confidence intervals (CI 95 %).

The models took into account only observations with no missing values for the different variables studied (‘complete case analysis’).

Analysis was performed using StataIC 11 software (StataCorp. 2011. Stata: Release 11. Statistical Software. College Station, TX: StataCorp LP).

## Results

### Uptake of healthcare

Of the 342 patients with HNC, 92.7 % had declared a referring doctor before cancer diagnosis. During the year preceding diagnosis, patients had consulted a health professional at least once in 87.7 % of cases and at least three times in 75.7 % of cases.

In 86.0 % of cases, patients had consulted a GP (Table 1). Amongst patients having consulted a GP, the median number of consultations was 5 [Q1 :3; Q3 :11]. As regards other health professionals, 21.1 % of patients had consulted a dentist, 47.1 % a non-ENT specialist, 16.4 % an ENT specialist and 43.0 % a nurse. The most consulted specialists outside of ENT were ophthalmologists (25.1 %) and specialists in cardiovascular pathology (14.0 %).

In the multivariable analysis (Table 2), consulting a GP at least once during the year preceding diagnosis was unrelated to Charlson index, age, sex, *département*, quintile of deprivation of place of residence. Consulting a dentist or a non-ENT medical specialist at least once during the year before diagnosis was associated with the deprivation quintile. Patients from the ‘quite privileged’, ‘quite underprivileged’ and ‘underprivileged’ quintiles consulted a dentist more frequently than those from the ‘very underprivileged’ quintile (*p* = 0.007). Patients from the ‘privileged’ quintile consulted a non-ENT specialist more frequently than those from the ‘very underprivileged’ quintile (*p* = 0.003). More frequent nurse visits were linked with the presence of 3 or more comorbidities (*p* = 0.011). Interactions between sex and age, and age and deprivation quintile were tested; they were not significant.

**Table 2:** Multivariable analysis of healthcare uptake (*N* = 340)

	General practitioner			Dentist			Non-ENT specialist			ENT specialist			Nurse		
	OR	CI 95 %	*p*	OR	CI 95 %	*p*	OR	CI 95 %	*p*	OR	CI 95 %	*p*	OR	CI 95 %	*p*
Sex			0.827			0.846			0.735			0.232			0.799
Male	1.00			1.00			1.00			1.00			1.00		
Female	1.09	0.47–2.55		0.93	0.46–1.89		1.11	0.62–1.98		1.54	0.76–3.11		1.08	0.59–1.97	
Age at diagnosis			0.162			0.070			0.081			0.739			0.520
< 55 years	1.00			1.00			1.00			1.00			1.00		
55–65 years	0.43	0.17–1.10		0.43	0.21–0.89		1.01	0.55–1.87		0.75	0.34–1.67		1.26	0.68–2.36	
> =65 years	0.72	0.22–2.35		0.52	0.21–1.28		2.07	0.96–4.45		0.96	0.35–2.67		1.56	0.73–3.36	
Charlson index			0.285			0.739			0.712			0.770			0.011
0	1.00			1.00			1.00			1.00			1.00		
1 to 2	2.12	0.69–6.54		1.08	0.43–2.69		1.21	0.54–2.75		0.95	0.35–2.57		1.87	0.74–4.74	
3 and over	2.99	0.77–11.60		0.81	0.27–2.45		1.47	0.56–3.84		0.70	0.20–2.44		4.06	1.41–11.7	
*Département*			0.249			0.109			0.769			0.701			0.261
Calvados	1.00			1.00			1.00			1.00			1.00		
Manche	1.39	0.51–3.74		0.81	0.30–2.15		0.95	0.43–2.09		1.15	0.43–3.11		1.16	0.52–2.59	
ZPL	2.36	1.00–5.56		1.98	0.91–4.28		1.30	0.68–2.46		0.88	0.37–2.08		1.27	0.66–2.46	
Somme	1.87	0.78–4.51		1.00	0.43–2.34		1.26	0.64–2.49		1.39	0.58–3.29		1.94	0.97–3.87	
Deprivation quintile			0.784			0.007			0.003			0.283			0.251
Privileged 1	1.41	0.47–4.20		1.67	0.64–4.31		3.70	1.67–8.18		1.22	0.46–3.22		1.95	0.92–4.12	
Quite privileged 2	1.39	0.49–3.95		3.21	1.33–7.78		1.33	0.66–2.71		1.54	0.61–3.87		2.04	0.98–4.22	
Quite underprivileged 3	0.70	0.27–1.83		3.14	1.25–7.89		0.71	0.34–1.52		0.32	0.07–1.48		1.45	0.67–3.12	
Underprivileged 4	1.12	0.47–2.69		3.73	1.76–7.90		0.75	0.40–1.38		1.60	0.74–3.46		1.27	0.68–2.38	
Very underprivileged 5	1.00			1.00			1.00			1.00			1.00		

Abbreviations: *OR* odds ratio, *CI* confidence interval, *ZPL* area around Lille, *ENT* ear, nose and throat

### Factors influencing stage at diagnosis

In univariate analysis (Table 1), a localised stage at diagnosis was more frequently associated with cancers of the oral cavity and larynx (*p* < 0.001), in patients consulting a dentist (*p* = 0.029) or an ENT specialist (*p* = 0.002) during the year prior to diagnosis.

Multivariable analysis of staging showed no association with sex, age at diagnosis, the Charlson index, the *département* or the deprivation quintile. Only tumour site was significantly associated with stage (*p* < 0.001) (result not shown).

**Fig. 1:**
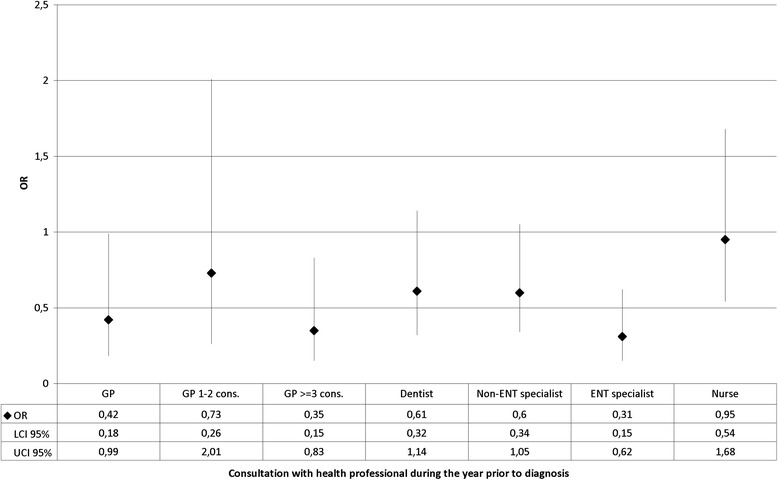
Factors associated with an advanced stage at diagnosis – multivariable analysis^a^. **a**. After adjustment for sex, age, Charlson index, department, tumour site and deprivation quintile. Abbreviation : OR = odds ratio,LCI = lower confidence interval, UCI = upper confidence interval, GP = general practitioner, ENT = ear, nose and throat

After adjustment for these variables, the stage was less advanced for patients who had consulted a GP (OR = 0.42 [0.18–0.99]) - with a dose–response effect when the number of consultations with the GP was divided into 3 categories (*p* = 0.022) - and also in those who had consulted an ENT specialist (OR = 0.31 [0.15–0.62]) (Fig. 1). For oral cavity cancers, seeing a dentist was not associated with stage at diagnosis (results not shown).

## Discussion

This study shows that patients developing HNC live in an underprivileged social environment in nearly two thirds of cases but that they are not excluded from the health system. Indeed, the vast majority of patients declared a referring doctor and consulted a GP during the year preceding their diagnosis, these consultations being regular in two thirds of patients. In addition, the act of seeking a GP consultation in this population is not socially determined and is associated with a diagnosis of localised cancer. As regards consultations with a dentist, this is rather infrequent, and the lower the socio-economic level of the place of residence is, the lower the rate of consultation is. There is no association between dentist consultation and a diagnosis of localised cancer.

With the exception of dysphonia in cancers of the larynx, most symptoms of HNC are non-specific but should be a cause for concern in patients with a high consumption of alcohol and tobacco. Each and every contact with a health professional should be an opportunity to make an early diagnosis of HNC and such opportunities are far from rare because in our study, 87.7 % of patients consulted a health professional at least once during the year prior to their diagnosis. This figure is close to that of Reid’s study [23].

A localised stage at diagnosis was related to consultation with a GP, with a dose–response effect according to the number of consultations. This dose–response effect suggests that medical monitoring has an impact on the stage at diagnosis. A similar result was found in a study carried out by Reid et al [28], on consultations with hospital physicians. This result needs to be considered in parallel with the fact that visits to the GP are frequent during the year prior to diagnosis. There is therefore a real potential for early diagnosis of these cancers by GPs in a target population, which remains to be defined.

Moreover, our results show that GP consultations are not linked to the deprivation index. This is all the more important considering that two thirds of the population studied live in underprivileged areas. In France, a country with universal healthcare coverage, patient payments for GP consultation are conjointly reimbursed by social insurance (roughly 70 %) and by complementary health insurance plans (around 30 %) if patients can afford them. Deprived patients are fully reimbursed by social insurance. Our results are thus generalizable only with countries with comparable health care organization. However, our results are consistent with other European studies showing that GP consultations, unlike specialist consultations, are not dependent on the socio-economic level [29].

In France, Dentists examine around 500 000 mouths a day [22]; initially, it would thus seem an obvious strategy to entrust early detection of oral cavity cancers to these health professionals. However, our study shows that the population of patients who developed HNC rarely visit the dentist (21.1 %). Coupled with this, dental consultations are socially determined : people living in the most affluent areas and those living in the most deprived areas consulted dentists least in the year preceding diagnosis. In the first case, we can hypothesise that the low rate of consultation is linked to generally good dental health requiring little care. In the second case, lack of access to dental care because of financial restraints might be suspected: in France, the most underprivileged patients forgo dental care 10.5 times more often than people who are not in a socially precarious position [30], and where dental care is not taken up, 49.9 % put forward financial reasons [31]. This lack of uptake is even more significant as 40.0 % of the population studied lived in the most deprived areas. What is more, our results show that HNC stage at diagnosis, particularly in the oral cavity, was not associated with dentist consultation in the year before diagnosis. Given that raising the awareness of these health professionals about early diagnosis of oral cavity cancers started only at the end of 2008, it may be that we have not yet had enough time to detect an effect. Altogether, in the absence of any scientific demonstration of a positive effect on the mortality rate, the pragmatic national policies on an HNC screening programme, based on dentists for early detection of oral lesions, risk having a deleterious effect on social inequalities in health care.

Finally, the association between a localised stage at diagnosis and consultation with an ENT specialist does not reflect the practice of early diagnosis of these cancers in the general population. It may be interpreted as the follow-up of various pathologies such as leucoplakia, erythroplakia or dysplastic lesions of the oral cavity and vocal cords, which can subsequently degenerate [32, 33].

The main strength of this study resides in the cross-analysis of data taken from cancer registries situated in high incidence areas and that from the national health insurance system. The study design was based on the method of ‘high-resolution population-based study’: data was collected in a precise and rigorous manner from the medical files in order to know all the characteristics of the cancer cases included. The inclusion of patients from the cancer registries allowed us to overcome the recruitment bias of hospital studies and give information on the totality of cancer cases in a given geographic area. Data taken from the national health insurance enabled us to identify all the health professionals consulted. This cross-analysis of nominative databases between the registries and the national health insurance is unusual because they do not operate on the same time frame. The cross-analysis of databases was carried out in January 2011 with the health insurance data which covered the period from 01.01.2009 to 31.12.2010 (data regarding utilization of healthcare are conserved for only two years). Thus, it was possible to have one full year of healthcare utilization data prior to diagnosis only for patients diagnosed in 2010. It was thus not possible to carry out a retrospective data collection regarding this uptake. Within the framework of this study, the registries tracked cases prospectively and validated HNC in priority in order to make the two time frames coincide.

Since information on the socioeconomic status of individuals is not available in cancer registries in France, the use of the deprivation index (EDI) is a pragmatic solution. Indeed, it is commonly argued that using area-level data is a valid and useful approach for circumventing the lack of individual information in medical files [33].

The main limit of our study is the small patient number, which precluded the possibility of completing a detailed topographical analysis of the tumours. It is true that our study covers the whole group of HNC whilst the recommendations for early diagnosis target oral cavity cancers only. Nevertheless, habits of medical care uptake concern the same at-risk population. The small number of patients limits the scope of our study. However, the study provides information on how patients recruited from four different cancer registries in a high-incidence area take up medical care. Lastly, data regarding uptake of healthcare are only available on patients registered under the general regime of the national insurance service but this covers 88 % of the French population [26]. Moreover, the patients unregistered (12 %) are affiliated to other public regimes. They have the same access to medical services and receive the same reimbursement rate than patients recorded to the general regime of the national insurance service. However, we don’t know the socio-economic status of this population.

The aim of this work was to describe patient habits in respect of utilization of medical services; it was therefore necessary to analyse only utilization before cancer diagnosis. However, as it was impossible to trace the specific medical consultation that began the cancer management, only medical services taken up between 12 months and 2 months before histological diagnosis of the cancer were taken into account.

## Conclusions

In view of the frequency of GP consultations, the existence of a significant association between GP consultations and a localised stage at diagnosis and the absence of a socio-economic aspect to this mode of medical services uptake, early detection of HNC by GPs would seem to be the most appropriate way. To this end, high-quality professional training for GPs is necessary. Nevertheless, the benefit of such early detection on the mortality rate of HNC remains to be shown and the target population must be defined.

## Abbreviations

ENT, Ear, Nose and Throat; ERASME, Extraction Research Analysis and Medico-Economic monitoring database; GP, general practitioner; HNC, head and neck cancers; ICD-O 3, International Classification of Diseases for Oncology, 3rd edition; IRIS, Ilots Regroupés pour l’Information Statistique; OR, Odds ratios; CI, Confidence Interval; Q1, 25th percentile; Q3: 75th percentile; ZPL, area around Lille
